# Native Entomopathogenic Fungi Against Whitefly (*Bemisia tabaci*) in Serrano Chili (*Capsicum annuum* L.)

**DOI:** 10.3390/insects17070680

**Published:** 2026-06-30

**Authors:** Magali Jiménez-Jiménez, Héctor Cabrera-Mireles, Alejandra Soto-Estrada, Lorena Jacqueline Gómez-Godínez, Felipe Gallardo-López, Jorge Jiménez-Zilli

**Affiliations:** 1 Colegio de Posgraduados Campus Veracruz, Manlio Fabio Altamirano 91690, Veracruz, Mexico; 2 Instituto Nacional de Investigaciones Forestales, Agrícolas y Pecuarias-Campo Experimental Cotaxtla, Medellín de Bravo 94276, Veracruz, Mexico; 3 Instituto Nacional de Investigaciones Forestales, Agrícolas y Pecuarias-Centro Nacional de Recursos Genéticos, Tepatitlán de Morelos 47600, Jalisco, Mexico; 4 Laboratorio de Biotecnología Andreb, Huatusco 94100, Mexico

**Keywords:** mycosis, culture medium, inoculation, whiteflies rearing, pest, chili seedlings

## Abstract

The whitefly (*Bemisia tabaci*) is a significant pest of chili crops, and the continuous use of insecticides to control it has led to insecticide resistance. This study aimed to characterize and evaluate the pathogenicity and virulence of native entomopathogenic fungus (EPF) strains from the Sotavento region against whiteflies in the laboratory. Fungi were isolated from whiteflies collected from chili plants in different habitats and identified through morphological and molecular characterization. The pathogenicity of nine EPF strains was assessed at 1 × 10^9^ spores mL^−1^, while the virulence of the most pathogenic strains was evaluated from 1 × 10^4^ to 1 × 10^9^ spores mL^−1^. The lethal concentration (LC_50_) and lethal time (LT_50_) were also determined. Two native genera of EPF were identified: *Beauveria* and *Cordyceps*. The strains JV01 and PV02 (*Cordyceps javanica*), SV01 (*Cordyceps fumosorosea*), and MV03 (*Beauveria bassiana*) demonstrated high pathogenicity, causing mortality rates ranging from 94% to 100% at a concentration of 1 × 10^6^ spores mL^−1^, five days after application. The strains JV01 and SV01 showed LC_50_ values of 1.65 × 10^5^ and 5.53 × 10^5^ spores mL^−1^, respectively, with an LT_50_ of 2 days for both. In conclusion, highly pathogenic and virulent native EPF strains were identified against adult whiteflies on serrano chili.

## Simple Summary

The whitefly (*Bemisia tabaci*) is a major pest of chili crops, and its management primarily relies on chemical insecticides. However, the continuous use of these chemicals has led to resistance issues and decreased effectiveness. In response to this challenge, the present study evaluated local entomopathogenic fungi as a safer, more sustainable alternative for controlling this pest, as they are already adapted to local environmental conditions. Fungi were collected from whiteflies found on chili plants, then isolated and identified. Their effectiveness was subsequently tested under laboratory conditions. The results indicated that four fungal strains had a high efficacy, causing mortality rates in whiteflies within two to five days. This suggests that native entomopathogenic fungi have the potential to serve as biological control agents for whiteflies. Utilizing these fungi could help reduce reliance on chemical insecticides, ultimately contributing to the sustainable management of chili crops.

## 1. Introduction

Mexico is the second-largest producer of green chili (*Capsicum annuum* L.) in the world, with 3,681,061 t, after China. In the state of Veracruz, it is the second most cultivated vegetable, with 5999.5 ha [1]. This crop is mainly affected by the whitefly (*Bemisia tabaci* Gennadius), an economically significant pest [2]. The whitefly reduces the photosynthesis rate in plants through the excretion of honeydew during feeding [3]; additionally, it transmits important viruses such as Begomoviruses and Criniviruses [4,5], which cause yellow leaf curling, leading to delayed plant growth and a significant reduction in fruit yield and quality [5,6]. Conventional pest management primarily relies on inorganic chemical insecticides, such as organophosphates, pyrethroids, and organochlorines [7]. However, their indiscriminate use has led to pest resistance, human toxicity, the death of beneficial insects and pollinators, and environmental contamination [8,9]; therefore, alternative strategies are needed to control whitefly populations.

Microbial control, particularly entomopathogenic fungi (EPF), is a viable alternative. Unlike other entomopathogenic agents, EPF do not need to be ingested by the insect to be effective [10]. They can directly infect insects through the cuticle, are host-specific, act as endophytes and antagonists of plant diseases, and are more cost-effective in the long term [11,12]. Among the genera of EPF found in the environment and utilized for pest control are *Metarhizium*, *Beauveria*, *Aschersonia*, *Entomophthora*, *Zoophthora*, *Erynia*, *Eryniopsis*, *Akanthomyces*, *Fusarium*, *Hirsutella*, *Hymenostilbe*, *Paecilomyces*, and *Verticillium* [13].

Using native EPF is crucial in pest control, as their adaptation to local conditions ensures virulence and persistence; moreover, their adaptation gives them an advantage over external strains [14,15]. The exploration and identification of pathogenic and virulent native EPF strains adapted to specific regions are essential for developing effective mycoinsecticides [16,17]. Despite the growing use of entomopathogenic fungi for whitefly control, limited information exists on native fungal strains adapted to tropical agroecosystems. Identifying locally adapted isolates with high pathogenicity and virulence is essential to improve biological control strategies and reduce dependence on synthetic insecticides. Therefore, this study aimed to isolate, characterize morphologically at the genus level, and molecularly at the species level, and evaluate the pathogenicity and virulence of native entomopathogenic fungal strains from the Sotavento region of Veracruz against adult *Bemisia tabaci* under laboratory conditions.

## 2. Materials and Methods

The study was conducted in two phases. The first phase involved collecting specimens of whiteflies infested with fungi from open-field chili crops, as well as from weeds surrounding the crops and in backyard areas, in a polygon with geographical coordinates: 18°50′ and 19°15′ N, 95°50′ and 96°25′ W, in the municipalities of Cotaxtla, Jamapa, Manlio Favio Altamirano, Medellín de Bravo, Paso de Ovejas, Puente Nacional, Soledad de Doblado, and Tlalixcoyan, all located in the Sotavento region of Veracruz, from August to December 2024. The second phase consisted of isolating, characterizing, and evaluating the pathogenicity and virulence of the collected fungi; this phase was carried out at the Entomology Laboratory of the National Institute of Forestry, Agricultural, and Livestock Research, at the Cotaxtla Experimental Field (INIFAP-Cotaxtla), Medellín, Veracruz.

### 2.1. Collection of Infested Whiteflies

The collection of these specimens was carried out directly in the field, following the method suggested by [18]. This process involved searching for whitefly corpses infected with fungi on the aerial parts of the plant. The collected corpses were placed individually in sterile plastic vials, which were labeled [19] for later isolation in the laboratory.

### 2.2. Isolation and Characterization

The collected specimens were disinfected in sodium hypochlorite (Industria Química del Istmo, S.A. de C.V., México city, México) (0.5% of the active product) for 5 min and rinsed three times with sterile distilled water, then cultured using the direct seeding technique [20] on 90 × 18 mm Petri dishes containing agar-dextrose-Sabouraud medium (SDA) (supplied by BD Bioxon^®^ Cuautitlán Izcalli, México state, México), supplemented with yeast extract (DIBICO^®^ supplied by DIBICO, SA DE CV, Cuautitlán Izcalli, México state, México) and the antibiotic enrofloxacin^®^ supplied by PiSA^®^ Farmacéutica, Coyoacán, Ciudad de México, México at a concentration of 1 mL L^−1^. The dishes with the inoculated specimens were incubated at 26 ± 2 °C with a relative humidity (RH) of 80 ± 5% until the colonies reached 2 to 4 cm in diameter. From these colonies, monosporic cultures were created to characterize the isolates, following [20]. This involved diluting the spores to 10^−2^, taking 10 µL, and placing them on agar-agar (DIBICO^®^), then incubating at 26 ± 2 °C for 24 h. After this period, a section of agar containing a single germinated spore was cut and transferred to a Petri dish with SDA medium to obtain a monosporic culture. Three repetitions were prepared for each isolate. The dishes were incubated at 26 ± 2 °C for 13 days [21]. Fungi were identified to the genus level through both macroscopic and microscopic characterization. Macroscopically, the shape, texture, margin, and color of both the front and reverse sides of each colony were recorded. For microscopic characterization, fungal tissue preparations were stained with lactophenol blue (supplied by Cientifica VelaQuin, Iztapalapa, Ciudad de México, México) and mounted on unstained, transparent Scotch tape to observe mycelial type, spore color, and spore shape. These characteristics were compared with taxonomic keys [22,23].

Based on the characteristics of the colonies, the frequency of isolates in each sampled area, and the literature consulted, nine strains were selected. The percentage of germination and colony diameter were estimated for these strains, which were assigned the codes MV01, MV02, MV03, JV01, JV02, PV01, PV02, SV01, and TV01 according to the origin of their collection sites (Table 1). For germination testing, the methodology of [24] was followed. From a concentration of 1 × 10^6^ spores mL^−1^, a sample of 200 µL was taken and deposited on agar-agar medium in Petri dishes. Three repetitions were performed for each strain. The inoculated dishes were incubated at 25 ± 2 °C for 24 h. After incubation, a drop of lactophenol was added to halt germination, and 200 germinated and non-germinated spores were counted per repetition. Spores were considered germinated when the germ tube was at least twice the diameter of the spore; this observation was made using a compound microscope with a 40x objective (model Red-220 MOTIC®, Xiamen, China). The colony diameter of monosporic isolates was recorded after 13 days of incubation [21].

### 2.3. DNA Extraction and Molecular Identification of Fungal Isolates

Molecular characterization was performed on isolates that excelled in the pathogenicity evaluation. Genomic DNA was obtained from the mycelium of previously cultured fungi using a modified version of the protocol described by [25]. DNA quantification was performed using a Thermo Fisher NanoDrop 2000 spectrophotometer (Thermo Fisher Scientific, Wilmington, DE, USA) [26,27]. To confirm the extraction, samples were run on 1% agarose gels at 80 V for one hour.

The ITS region of the fungal DNA was amplified by PCR [28,29] using the universal primers ITS1 and ITS4 [30,31] and was used as a primary approach for fungal identification. The PCR conditions consisted of an initial denaturation at 95 °C for 3 min, followed by 35 cycles of denaturation at 95 °C for 30 s, annealing at 55 °C for 30 s, and extension at 72 °C for 1 min, with a final extension step at 72 °C for 7 min. The presence of the amplified fragments was verified again on 1% agarose gels [32]. The samples were subsequently purified using a commercial magnetic bead system [33,34]. Sanger sequencing was performed on an ABI 3500 sequencing system using the service provided by Macrogen [35].

The bidirectional reads for the ITS gene were assembled into contigs and cleaned to generate consensus sequences in FASTA format using BioEdit. These sequences were compared with the Nucleotide database using the NCBI BLASTn tool, (National Center for Biotechnology Information, Bethesda, MD, USA; https://blast.ncbi.nlm.nih.gov/Blast.cgi, accessed on 4 April 2026), with match coverage ranging from 90% to 100%. Reference ITS sequences used for phylogenetic comparisons were retrieved from GenBank based on sequence similarity, taxonomic identity, and sequence quality. Information regarding the reference sequences used in the phylogenetic analyses, including GenBank accession numbers, taxonomic identification, and corresponding ITS sequences. For subsequent analyses, independent sequences were aligned using the ClustalW algorithm from BioEdit and then concatenated [36]. Phylogenetic reconstruction was performed in MEGA12 using the maximum likelihood method, applying the GTR evolutionary model with gamma distribution and 1000 bootstrap replicates [37]. The final cladogram was edited and visualized in FigTree (v1.4.4.). Finally, the obtained sequences were uploaded to NCBI with the ID SUB16150138.

### 2.4. Management of Whiteflies

**Figure 1:**
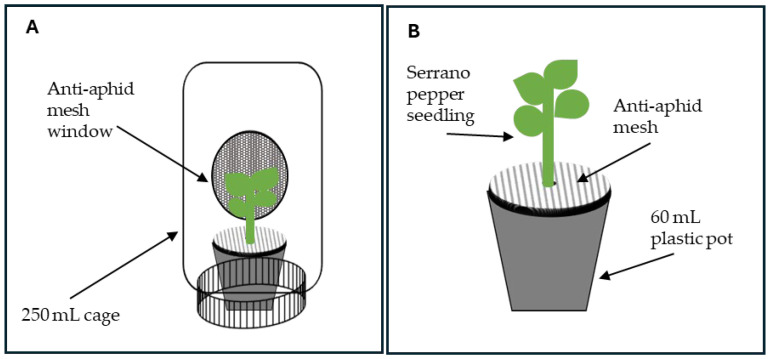
Cage design for assessing the pathogenicity and virulence of fungi isolated from whiteflies. (**A**) Cage with window, (**B**) covered with anti-aphid mesh over the cups.

Whitefly (*B. tabaci*) breeding was established with adults collected in serrano pepper plantations in the Sotavento region, Veracruz, where entomopathogenic fungi had never been applied. The insects were kept in entomological cages (40 × 40 × 40 cm) covered with anti-aphid mesh, which contained serrano pepper plants as a food source, under greenhouse conditions. Environmental conditions were controlled at 28–32 °C, 60–80% relative humidity, and a 12:12 h (light:dark) photoperiod. After five generations, the adult whiteflies were used for bioassays. For the experiment, 250 mL plastic bottles were used as cages, designed with a circular side window (5 cm diameter) covered with anti-aphid mesh (Figure 1A). Inside each bottle, 60 mL plastic cups (6.9 cm wide by 8.6 cm high) containing two-month-old serrano chili seedlings planted in peat moss substrate were placed. The cups with the seedlings were covered with anti-aphid mesh at the base of the stem (Figure 1B), to prevent dead flies from becoming lost in the substrate.

### 2.5. Pathogenicity Assessment

For this test, the nine strains indicated in the germination test were used. For each strain, a spore suspension of 1 × 10^9^ mL^−1^ was prepared, dissolved in sterile distilled water with 0.1% Tween^®^ 80 (Sigma-Aldrich Química, Toluca, México). Each strain corresponded to a treatment; a control was also included, consisting of sterile distilled water with 0.1% Tween 80, resulting in a total of 10 treatments, distributed in a completely randomized design with three repetitions (three replicas per repetition). Each cage was treated as an experimental unit. Three days before spore inoculation, 10 adult whiteflies from the breeding stock were placed in each cage with serrano chili plants to allow them to adapt to the cage conditions. The spore application involved administering a 0.1 mL spray on each plant using a 20 mL manual sprayer. The treated experimental units were maintained at 26 ± 2 °C, 80 ± 5% RH, and a 12:12 (light:dark) photoperiod. Mortality was recorded every 24 h for 5 days after application (DAA). The dead flies were removed from the cages and cultured on SDA medium to confirm their infection by the applied fungus.

### 2.6. Virulence Assessment

Based on the pathogenicity results, four outstanding strains (MV01, JV01, PV02, and SV01) were selected for virulence evaluation, and the lethal concentration (LC_50_) and lethal time (LT_50_) were determined. For each strain, five concentrations were evaluated: 1 × 10^4^, 1 × 10^6^, 1 × 10^7^, 1 × 10^8^ and 1 × 10^9^ spores mL^−1^; the spores were mixed in sterile distilled water with 0.1% Tween 80. The control consisted of sterile distilled water with 0.1% Tween 80. A total of 21 treatments were conducted, distributed in a completely randomized design with three repetitions (three replicas per repetition). The spore inoculation method was the same as that used in the pathogenicity assessment. The response variable was the percentage of mortality recorded every 24 h for 5 days. The dead whiteflies were removed from the cages and cultured on SDA medium to confirm their infection by the entomopathogenic fungus.

### 2.7. Statistical Analyses

Descriptive statistics were applied to morphological characterization data; specifically, colony diameter was evaluated using an analysis of variance (ANOVA). Mortality data associated with pathogenicity and virulence were corrected with the Abbott formula [38] and subsequently analyzed using the nonparametric Kruskal–Wallis test at a significance level of *p* = 0.05, using InfoStat version 2020 software. LC_50_ and LT_50_ were estimated using Probit regression using Statgraphics Centurion v19.

## 3. Results

### 3.1. Collection and Isolation

A total of 332 fungal strains were isolated from whiteflies; of these, 283 were collected from serrano, habanero, and jalapeño pepper plantations, 5 from backyard plants of chiltepín and chilpaya, and 44 from weeds (Table 2). During the isolation process, it was observed that mycosis occurred in both nymphs and, particularly, in adults.

### 3.2. Macroscopic and Microscopic Characteristics

**Figure 2:**
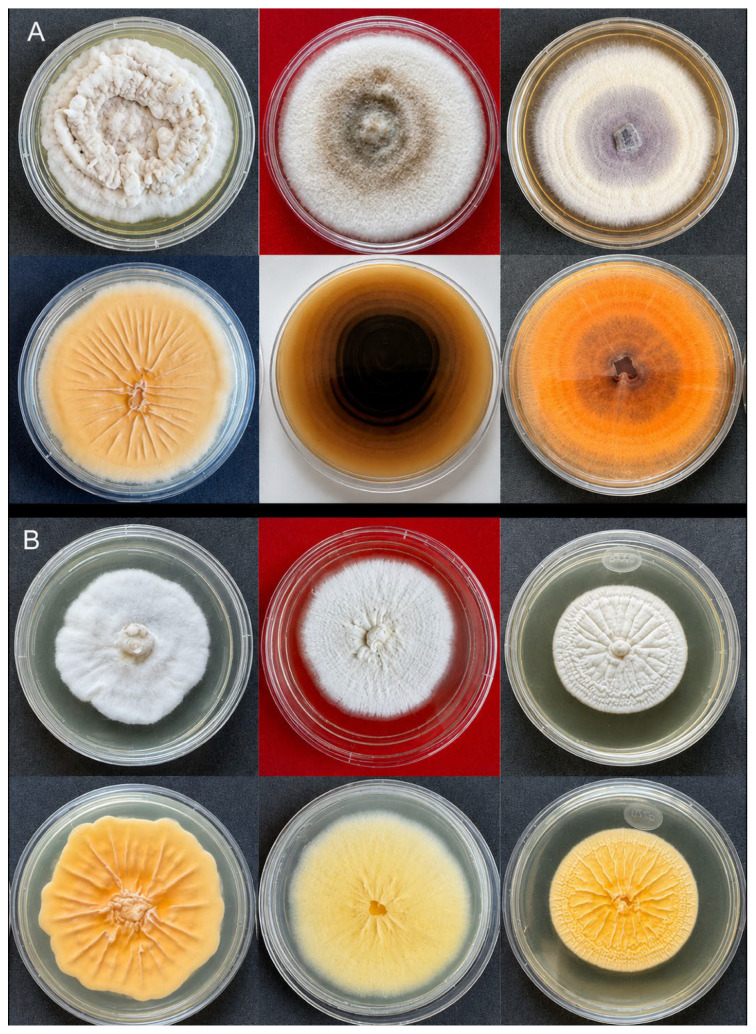
Macroscopic characteristics of the front and back of fungal strains isolated from mycosed whiteflies in the Sotavento region, Veracruz. (**A**) *Cordyceps* spp., (**B**) *Beauveria* spp.

Based on the characteristics of the isolates, 193 corresponded to *Cordyceps* spp. and 139 to *Beauveria* spp. Greater variability was observed in the macroscopic characteristics of the *Cordyceps* genus. In the colonies of this genus, circular growth was observed, with elevated white mycelium that turned pinkish as it developed, a central indentation, and a fluffy, granular, powdery texture. The reverse of the plate exhibited cream-colored growth with distinct rays. Flat circular colonies of a pinkish color were also observed, characterized by more intense pink concentric rings, a fluffy texture, and a cream reverse with prominent brown rings. Additionally, flat circular colonies with white rings, a lilac center, a fibrous texture, and a cream reverse with well-defined rings were noted. Other colonies turned white, displaying a light gray basipetal center, a prominent fluffy edge, and a cottony-powdery texture, with a cream reverse (Figure 2A).

In contrast, the *Beauveria* isolates exhibited fluffy, circular colonies ranging from white to cream, with a slight central elevation, a fibrous texture, irregular edges, and a cream reverse. Some colonies appeared flat and white, with rings, a powdery texture, and a cream reverse. Additionally, slow-growing colonies of creamy white color, with a cottony-woolly texture, concentric rays, well-defined edges, and a cream reverse with distinct rays were observed (Figure 2B).

**Figure 3:**
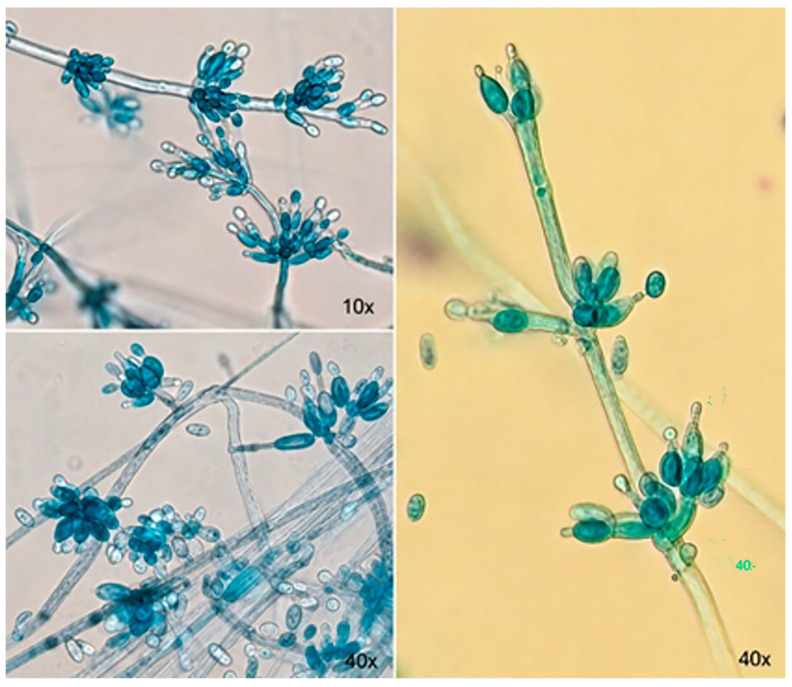
Microscopic characteristics of fungal strains of the *Cordyceps* spp. genus isolated from mycosed whiteflies in the Sotavento region, Veracruz.

Regarding the microscopic characteristics, the *Cordyceps* isolates exhibited septate, branched mycelium, along with branched conidiophores terminating in clusters of subglobose phialides arranged in whorls with a narrow neck, from which oval to elliptical, unicellular conidia were formed in chains (Figure 3).

**Figure 4:**
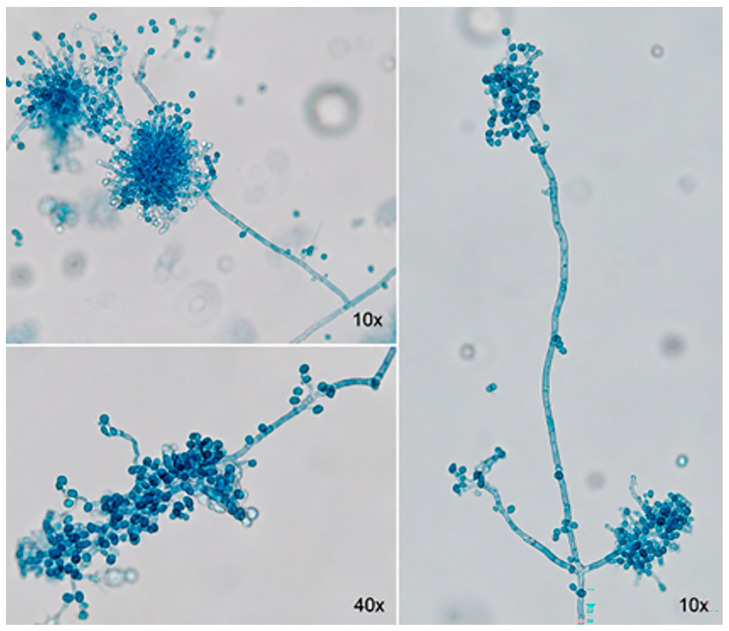
Microscopic characteristics of *Beauveria* spp. fungal strains isolated from mycosed whiteflies in the Sotavento region of Veracruz.

On the other hand, the *Beauveria* isolates exhibited septate mycelium and short, branched conidiophores that emerged from the main hyphae. These conidiophores produce phialides with a wide base and an elongated neck, arranged in dense clusters. A rachis extends in a zigzag pattern from the conidiogenous cells, forming small, unicellular, hyaline, globose conidia (Figure 4).

The germination percentages for the strains ranged from 72.7% to 82.6%. Strains JV02, PV01, PV02, and SV01 exhibited spore germination rates above 80%, while strains MV01, MV02, MV03, TV01, and JV01 displayed germination rates between 72% and 78% (Table 3). Regarding colony diameter, a highly significant difference was observed (F_8,18_ = 15.85, *p* = 0.001). Strains JV01, JV02, and SV01 of *Cordyceps* showed greater mycelial growth (ranging from 71.6 to 83.0 mm) compared to strains MV01, MV02, MV03, and TV01 of *Beauveria* (ranging from 52.8 to 58.5 mm) (Table 3).

**Table 3:** Spore germination and colony diameter of fungal strains isolated from whiteflies in the Sotavento region of Veracruz.

Code	Germination (%)	Colony Diameter (mm)
JV01	78.07	83.08 a
JV02	80.98	73.96 a
MV01	73.97	58.48 bc
MV02	74.47	58.57 bc
MV03	72.74	57.52 c
PV01	82.64	73.54 a
PV02	81.18	71.63 ab
SV01	82.09	73.26 a
TV01	73.23	52.81 c

Means within columns with the same lowercase letters are not significantly different from each other (*p* > 0.05).

### 3.3. Molecular Identification of Fungal Isolates

**Figure 5:**
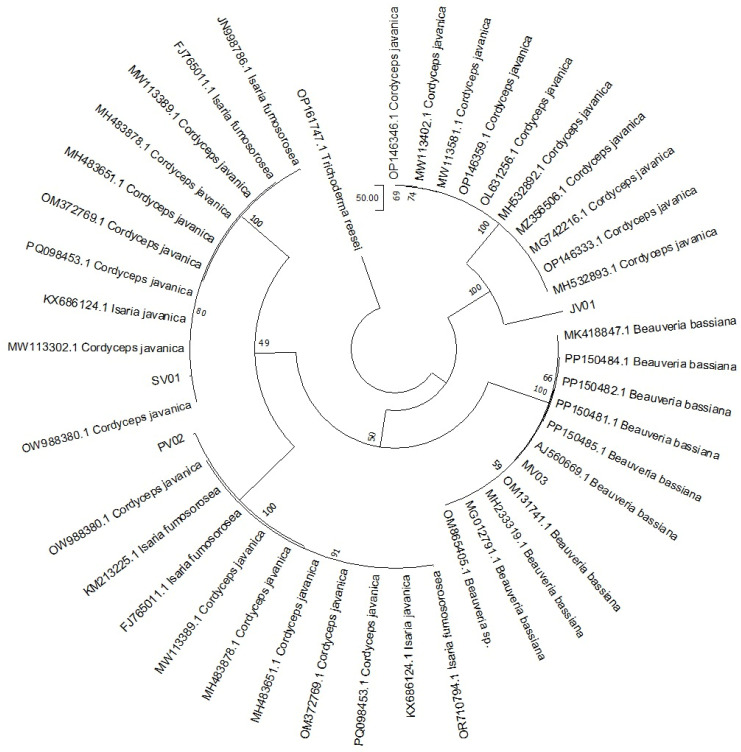
Phylogenetic tree of the four strains used in this report. Gene (ITS) Maximum Likelihood tree of the four strains. Each number represents bootstrap support values from 1000 replicates, as indicated at the nodes. The root of this tree was the fungus OP161747.1_*Trichoderma reesei*.

In addition to morphological and microscopic characterization, molecular characterization was performed on the four isolates that exhibited the highest pathogenicity. Species assignments were primarily based on ITS sequence analysis; however, definitive species identification would require additional morphological assessments and/or multilocus analyses. The results showed that isolates JV01 and PV02 clustered within a clade corresponding to *Cordyceps* sp. (ITS-affiliated with *C. javanica*), with sequence identity values ranging from 90% to 98%. Isolate SV01 clustered with *Cordyceps* sp. (ITS-affiliated with *C. fumosorosea*), showing 95% sequence identity, whereas isolate MV03 corresponded to *Beauveria* sp. (ITS-affiliated with *B. bassiana*), with 98% sequence identity (Figure 5).

### 3.4. Pathogenicity of the Strains

**Figure 6:**
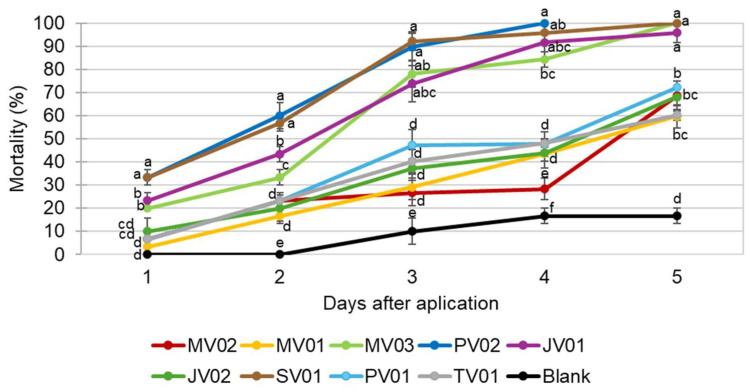
Pathogenicity of the nine strains on the whitefly, applied at a concentration of 1 × 10^9^ spores mL^−1^. Means followed by the same letter are not significantly different (*p* > 0.05).

A significant statistical difference was observed between strains at a concentration of 1 × 10^9^ spores mL^−1^ regarding whitefly mortality over the five evaluated days; day 1 (H = 22.81, *p* = 0.004), day 2 (H = 24.59, *p* = 0.002), day 3 (H = 25.35, *p* = 0.002), day 4 (H = 25.97, *p* = 0.001), and day 5 (H = 25.08, *p* = 0.001). Over the five days of evaluation, strains PV02, SV01, MV03, and JV01 stood out for causing the highest mortality rates. It was observed that at 4 DAA, PV02 caused 100% mortality of whiteflies. Strains SV01 and MV03 achieved 100% mortality at 5 DAA, followed by JV01 with 96%. The control group remained below all strains, showing a mortality rate of 19.0% (Figure 6).

### 3.5. Virulence of the Strains

**Figure 7:**
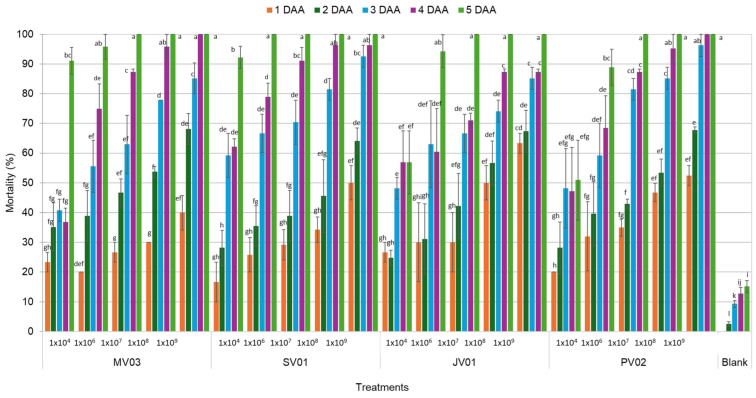
Virulence of the strains at different concentrations on whiteflies, recorded over five days after application. DAA = Days after application. Means followed by the same letter are not significantly different (*p* > 0.05).

Regarding virulence, factorial analysis indicated significant differences between strains, concentrations, and days (H = 364.05, *p* = 0.0001). The different strains showed an increase in mortality over time, associated with higher concentrations. All strains successfully induced mycosis from 3 DAA to 5 DAA. Concentrations of 1 × 10^6^, 1 × 10^7^, 1 × 10^8^, and 1 × 10^9^ spores mL^−1^ were particularly effective in causing higher mortality percentages, in contrast to the concentration of 1 × 10^4^ at 5 DAA. The four evaluated strains demonstrated virulence in controlling the whitefly population; however, strains JV01, MV03, and SV01 were the most efficient, causing mortalities of 94.3%, 95.8%, and 100%, respectively, at 5 DAA with a concentration of 1 × 10^6^. Strain PV02 caused 100% mortality at a concentration of 1 × 10^7^ at 5 DAA. The control group showed a maximum mortality of 15% was observed at the end of the experiment (Figure 7).

### 3.6. Estimation of LC_50_ and LT_50_

The estimated regression equations showed an optimal fit, with R^2^ values ranging from 0.91 to 0.97 (*p* < 0.01) and highly significant χ^2^ values (*p* < 0.01). This confirms a clear linear dose–response relationship between dose increment and mortality. The JV01 and SV01 strains were the most effective as they had the lowest LC_50_ (1.65 × 10^5^ and 5.53 × 10^5^ mL^−1^ spores, respectively) and LT_50_ of 2.2 and 2.0 days. On the contrary, the MV03 and PV02 strains showed the highest LC_50_ values and the longest LT_50_ (Table 4).

**Table 4:** Lethal concentration (LC_50_) and lethal time (LT_50_) of the evaluated strains.

Strain	Regression Equation	* *R*^2^	* *χ*^2^	LC_50_ (Spores mL^−1^)	LT_50_ (Days)
				Interval of Confidence 95%	Interval of Confidence 95%
MV03	Y = 5.4831 + 0.2167 X	0.97	111.05	3.69 × 10^6^ (2.70 × 10^6^–5.03 × 10^6^)	2.71 (1.32–5.56)
SV01	Y = 4.2929 + 0.2899 X	0.96	86.41	5.53 × 10^5^ (3.47 × 10^5^–8.83 × 10^5^)	2.02 (1.23–3.32)
JV01	Y = 3.8747 + 0.2683 X	0.95	46.88	1.65 × 10^5^ (9.72 × 10^4^–2.79 × 10^5^)	*2.23 (1.23–4.02)*
PV02	Y = 2.5832 + 0.7353 X	0.91	86.10	1.82 × 10^6^ (2.63 × 10^5^–1.26 × 10^7^)	4.02 (2.87–5.65)

*R*^2^: good fitness, *χ*^2^: calculated value of chi-square, *: *p* values < 0.01.

## 4. Discussion

The macroscopic characteristics of the strains of the genus *Cordyceps* recorded in this research are consistent with those reported by [39], who observed colonies of *Cordyceps* cultivated in SDA exhibiting a pale pink color and concentric growth. Similarly, Ref. [40] noted that white colonies of *C*. *fumosorosea* transitioned to a smoky pink 15 days post-incubation. In contrast, the characteristics of the *Beauveria* strains align with those observed by [41], who documented cottony white colonies that later developed a creamy and dusty appearance. Ref. [42] also observed white cottony mycelium colonies with a semi-elevated surface.

Regarding the microscopic characteristics of *Cordyceps*, the formation of phialides and conidia matches the descriptions provided by [43], who noted phialides with a globose and broad basal portion, along with a long distal neck. Ref. [44] highlighted the presence of oval-shaped conidia originating from successively formed phialides, which develop into chains. Likewise, Ref. [45] reported phialides possessing a broad globose basal portion, a long distal neck, and ellipsoidal conidia. The characteristics of *Beauveria* identified in this research agree with those described by [21], who noted broad conidiogenous cells with a globose base from which conidia emerge, as well as a zig-zag-shaped rachis. The identification and isolation of various fungal strains is a crucial step in the development of mycoinsecticides or biocontrol agents for pest management [46].

Molecular identification of the isolated entomopathogenic fungal strains, through sequencing of the ITS region, confirmed their membership in well-established genera such as *Cordyceps* and *Beauveria*. Strains JV01 and PV02 showed 90% to 98% genetic identity with *Cordyceps javanica*, strain SV01 confirmed 95% identity with *Cordyceps fumosorosea,* while strain MV03 achieved 98% identity with *Beauveria bassiana*. These identities were confirmed by phylogenetic analysis, which showed that the strains group appropriately within their respective genera. The molecular analysis provided crucial support for the previously obtained morphological and microscopic results. The application of Sanger sequencing and phylogenetic analysis demonstrated that molecular techniques are key to this type of study [47,48].

The germination percentage of spores ranged from 72.7 and 82.6%, comparable to those reported by [49], who observed germination rates of 60 to 100% in strains of *B. bassiana*. In contrast, Ref. [50] reported conidial germination rates of 93 to 98.9% for strains of this species and 97 to 98.3% for strains of *C. fumosorosea*. Ref. [51] emphasized that efficient germination of EPF spores is a critical characteristic for ensuring their effectiveness as biological pest control agents. Furthermore, variability was observed in the mycelial growth capacity across the two fungal genera; however, the strains of the genus *Cordyceps* exhibited the largest colony diameters (71.6 to 83.0 mm), values similar to those reported by [52] for this genus (80.1 to 81.0 mm). In contrast, Ref. [53] had colony diameters of 53.3 and 69 mm in *C. farinosa* and *C. fumosorosea*, respectively, at a temperature of 25 °C.

The pathogenicity results demonstrated the high efficacy of four strains in causing mortality in whiteflies. Notably, the strain PV02, belonging to the genus *Cordyceps*, achieved 100% mortality at 4 DAA, highlighting its speed and effectiveness. This outcome significantly surpasses the findings of [15], who reported that strains of *Isaria* (reclassified as *Cordyceps javanica*) resulted in 80% mortality after 7 days. Similarly, the strains SV01 and JV01 also demonstrated pathogenic potential, reaching 100% and 96% mortality, respectively, at 5 DAA. Furthermore, the strain MV03 of the genus *Beauveria* caused 100% mortality of whiteflies at 5 DAA, which greatly exceeded the findings reported by [54], who observed whitefly mortalities ranging from 71.67% to 98.33% at 3 and 9 days with strains of *B. bassiana*, while [55,56] reported values below 30% and 50%, respectively, with strains of the same species. The pathogenicity test is a fundamental indicator for measuring the efficacy of pathogenic fungi against pests, as it directly assesses their capacity to infect and cause disease in the host insect [57]. Fungal strains that exhibit high pathogenicity are considered promising candidates for application as biological control agents [58]; this is the case for the evaluated strains that were effective in this research.

Regarding virulence, the four strains demonstrated the ability to cause mortality between 3 and 5 DAA, confirming their potential as entomopathogenic agents. The JV01 and SV01 strains of the genus *Cordyceps*, as well as the MV03 strain of the genus *Beauveria*, at a concentration of 1 × 10^6^ spores mL^−1^, achieved mortality rates ranging from 94% to 100% at 5 DAA, indicating their high virulence. The PV02 strain of the genus *Cordyceps* required a higher concentration (1 × 10^7^ spores mL^−1^) to cause 100% mortality at 5 DAA. This contrasts with the findings of [15], who reported that a higher concentration (1 × 10^7^ spores mL^−1^) of *Isaria javanica* was needed to achieve mortality rates of 75% to 80% at 7 DAA. In the case of *B. bassiana*, Ref. [59] reported that a higher concentration (1 × 10^9^ spores mL^−1^) was necessary to achieve 100% mortality in adult whiteflies at 6 DAA.

The LC_50_ and LT_50_ results indicated that the strains JV01 and SV01 (*Cordyceps* spp.) were efficient in achieving LC_50_ at low concentrations (1.65 × 10^5^ and 5.53 × 10^5^ spores mL^−1^, respectively) with an LT_50_ of 2 days in both cases. These values contrast with those reported by [60], who determined an LC_50_ at a concentration of 1 × 10^7^ spores mL^−1^ and an LT_50_ ranging from 12 to 20 days. In this study, the strains MV03 (*Beauveria*) and PV02 (*Cordyceps*) exhibited LC_50_ values of 3.69 × 10^6^ and 1.82 × 10^6^ spores mL^−1^. These values contrast with those reported by [60], who determined an LC_50_ of 1 × 10^7^ spores mL^−1^ and an LT_50_ of 12 to 20 days. In this study, the strains MV03 (*Beauveria*) and PV02 (*Cordyceps*) exhibited LC_50_ values of 3.69 × 10^6^ and 1.82 × 10^6^ spores mL^−1^ respectively, with LT_50_ values between 2.7 and 4 days. These results indicate that these strains can be considered virulent, as suggested by similar studies, particularly those involving *Beauveria* strains, with LC_50_ values ranging from 0.22 × 10^4^ to 4.91 × 10^6^ spores mL^−1^ and LT_50_ values of 3.0 and 4.0 days [61]. Likewise, [55] identified a virulent strain of *B. bassiana* with an LC_50_ of 0.92 × 10^6^ spores mL^−1^ and LT_50_ of 6.10 to 11.66 days. Virulence is a critical attribute for selecting highly effective strains for biological pest control using EPF, so the accurate estimation of parameters such as LC_50_ and LT_50_ is essential, given the varying degrees of virulence among strains within the same species [55,61]. Furthermore, Ref. [54] notes that the viability of spores should also be considered when evaluating the performance of a strain.

## 5. Conclusions

Four strains of native entomopathogenic fungi were identified: JV01 and PV02 as *Cordyceps javanica*, SV01 as *Cordyceps fumosorosea*, and MV03 as *Beauveria bassiana*, all of which were highly pathogenic. In particular, the strains JV01 and SV01 emerged as the most promising due to their high virulence, displaying the lowest LC_50_ values (1.65 × 10^5^ and 5.53 × 10^5^ spores mL^−1^, respectively) and presenting an LT_50_ of only 2 days in both cases. These characteristics make them excellent candidates for controlling the whitefly *Bemisia tabaci* in serrano chili.

## Figures and Tables

**Table 1:** Origin of the strains collected in the Sotavento region, Veracruz, México.

Code	Habitat/Host	Genus	Location
MV01	Backyard/chilpaya chili	*Beauveria* spp.	Medellín
MV02	Plantation/habanero chili	*Beauveria* spp.	Medellín
MV03	Plantation/habanero chili	*Beauveria* spp.	Medellín
JV01	Arvense	*Cordyceps* spp.	Jamapa
JV02	Arvense	*Cordyceps* spp.	Jamapa
PV01	Plantation/serrano chili	*Cordyceps* spp.	Paso de Ovejas
PV02	Plantation/serrano chili	*Cordyceps* spp.	Paso de Ovejas
SV01	Arvense	*Cordyceps* spp.	Soledad de Doblado
TV01	Arvense	*Beauveria* spp.	Tlalixcoyan

**Table 2:** Fungal strains obtained from mycosed whiteflies collected from several habitats of the Sotavento region, Veracruz.

Municipality	Code	Habitat		
		Plantation	Backyard	Arvense
Cotaxtla	CV01-CV47	33		14
Jamapa	JV01-JV11			11
Medellín De Bravo	MV01-MV209	196	5	8
Paso de Ovejas	PV01-PV18	18		
Soledad De Doblado	SV01-SV08	2		6
Tlalixcoyan	TV01-TV08	3		5
Puente Nacional	PNV01-PNV31	31		
Total		283	5	44

Blank spaces mean that no whiteflies infected by EPF were found.

## Data Availability

Data sharing does not apply to this article. The datasets used in this article are not available because they are part of a patent translation process.
